# Mapping surface charge density of lipid bilayers by quantitative surface conductivity microscopy

**DOI:** 10.1038/ncomms12447

**Published:** 2016-08-26

**Authors:** Lasse Hyldgaard Klausen, Thomas Fuhs, Mingdong Dong

**Affiliations:** 1 Interdisciplinary Nanoscience Center (iNANO), Aarhus University, Gustav Wieds Vej 14, 8000 Aarhus C, Denmark; 2 Department of Pharmacy, Faculty of Health and Medical Sciences, University of Copenhagen, Universitetsparken 2, Copenhagen Ø DK-2100, Denmark

## Abstract

Local surface charge density of lipid membranes influences membrane–protein interactions leading to distinct functions in all living cells, and it is a vital parameter in understanding membrane-binding mechanisms, liposome design and drug delivery. Despite the significance, no method has so far been capable of mapping surface charge densities under physiologically relevant conditions. Here, we use a scanning nanopipette setup (scanning ion-conductance microscope) combined with a novel algorithm to investigate the surface conductivity near supported lipid bilayers, and we present a new approach, quantitative surface conductivity microscopy (QSCM), capable of mapping surface charge density with high-quantitative precision and nanoscale resolution. The method is validated through an extensive theoretical analysis of the ionic current at the nanopipette tip, and we demonstrate the capacity of QSCM by mapping the surface charge density of model cationic, anionic and zwitterionic lipids with results accurately matching theoretical values.

## Abstract (web summary)

Surface charges on lipid bilayers deeply influence the way proteins interact with cellular membranes, yet their precise quantification has proven challenging. Here, the authors report on a quantitative method to map and evaluate surface charge densities under physiological conditions.

## Graphical abstract

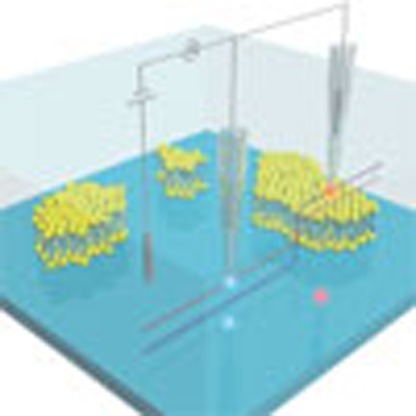

Surface charges can be found at virtually any water submersed surface, especially on biological exteriors. They arise from interactions with the solvent, leading to the dissociation or adsorption of molecules with an ionic charge^1^. An intricate electrical field is created by the surface-bound ions, and the sign and density of surface charges define the properties of the surface. The surface charge of cell membranes differs between species^2^, and the complex composition of mammalian cell membranes creates a spatially heterogeneous structure with different combinations of lipids and proteins in domains^3^,^4^, for example, in lipid rafts, where the charge of the lipid head groups is important for protein uptake and for cell signalling^5^. Artificial membranes, mimicking the true membrane composition, can be routinely produced with a large range of lipids, and experiments with lipids exhibiting different electrostatic charge have shown that uptake of specific proteins can be enabled simply by tuning the ratio of the lipids^6^. Modifying the charge of liposomes has also proven to be important in gene delivery to increase transfection rates^7^, and in drug delivery to prevent the absorption of blood proteins^8^ that would otherwise lead to immune recognition.

Structural information about lipid bilayers can be obtained from a range of spectroscopy methods^9^, while spatial variations in the lipid composition is determined by fluorescence microscopy with specially designed fluorophores^10^ or scanning probe methods measuring small changes in mechanical properties^11^. The surface charge density (SCD) of lipid bilayers is however not simply a function of the lipid composition, it also depends on environmental factors such as pH, temperature and salt concentration^12^. The salt concentration becomes problematic as the high ionic strength under physiological conditions screens the electrical field with a Debye length of less than a nanometre. As a consequence, mapping of the SCD of lipid bilayers is mainly based on simulations or *ex situ* methods such as Kelvin Probe^13^, colloidal probe^14^ or the D-D mapping method in atomic force microscopy (AFM)^15^ performed at low electrolyte concentration. The preferred way of determining average SCDs of lipid structures is through experiments based on electrokinetic effects, such as electrophoresis of suspended liposomes or streaming potential measurements of supported bilayers^16^. These methods are used on bulk structures, but the electrokinetic properties are preserved for nanoscale lipid structures, and a method based on these effects could be an ideal setup for mapping the SCD of lipid bilayers.

Scanning ion-conductance microscopy (SICM)^17^ is a unique type of scanning probe technique that uses an electrolyte filled nanopipette to measure the topography of samples, mainly living cells, submerged in an electrolyte bath. An electrical bias potential applied between an electrode inside the pipette and an electrode in the bath drives a net ionic current through the pipette tip. As the pipette is approached to a non-conductive sample, the resulting occlusion of ion flow can be used for distance feedback. The unique feedback mechanism enables non-contact imaging, and several studies have underlined the advantages compared with the traditional tapping mode AFM^18^,^19^,^20^. The lateral and vertical resolution of SICM is influenced by the size of the nanopipette^21^, and subnanometre vertical resolution has previously been attained with small pipettes^22^,^23^, while the lateral resolution is limited to around two times the tip inner radius^24^ corresponding to 30 nm in this study. The image formed by SICM is not necessarily an accurate representation of the sample topography, as surface charges influence the measured topography. This happens as SICM topography is obtained under the assumption of uniform solution conductivity. The effect of surface charge on the local conductivity at physiological conditions has long been believed to be much smaller than the practical resolution^25^, but several recent studies have suggested that this is a misconception^26^,^27^. A bias dependent accumulation of counter ions at the pipette aperture, in combination with the asymmetric tip geometry, gives rise to a non-linear potential-current relationship^28^,^29^,^30^. The effect of this phenomenon has recently been investigated using two different modes, a distance-modulated mode, where the pipette is vertically oscillated near the sample surface at a constant potential^31^, and a bias-modulated mode, where the pipette is approached to the surface and cyclic voltammetry performed^32^. A phase-shift in the current was observed in both methods, which was believed to be associated with the accumulation of counter ions at the sample surface and a non-trivial time constant of ionic mass-transport. Both studies showed qualitative measurements of surface charge, in the sense that an area of high-charge density was identified from an area of low-charge density, but further quantitative measurement was not achieved.

In this work we propose a new approach capable of quantitatively measuring the SCD with a spatial resolution comparable with the traditional SICM. In the new approach, developed simultaneously to the above mentioned studies, the topography error occurring due to surface conductivity is investigated by scanning the same topography with different bias potentials. The ionic current at the nanopipette tip is investigated through a careful Poisson–Nernst–Planck (PNP) analysis, and a height-correction routine capable of extracting the true topography from SICM images is established. A new mode of SICM, quantitative surface conductivity microscopy (QSCM), is furthermore described. This method is capable of mapping surface charge densities under physiological conditions, presenting a significant scientific improvement. The lateral resolution is around 30 nm based on the pipette sizes used in this study, which is high enough to resolve typical lipid rafts. Experimental proof of the new method is given by the imaging of three different lipid bilayers and characterization of three homogeneous substrates of distinct SCD. Results show an excellent agreement with theoretical SCDs calculated using the Gouy–Chapman–Grahame–Stern model^33^ and the extended Poisson–Boltzmann approximation^34^.

## Results

### Characterization of supported lipid bilayers by SICM

**Figure 1:**
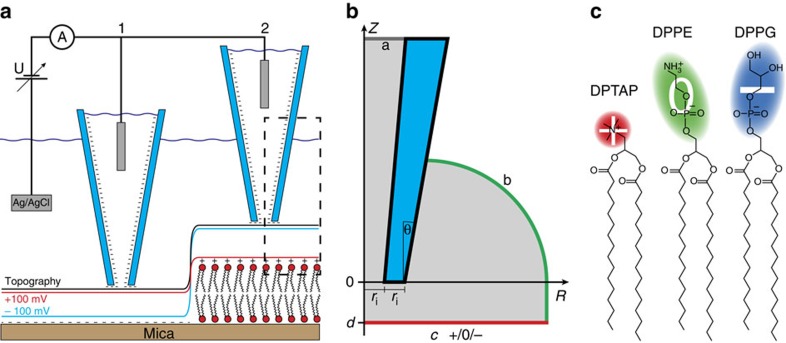
Schematic of the experiment. (**a**) Schematic of the SICM setup and QSCM mode. A bias is applied between the Ag/AgCl electrodes inside the pipette and in the bath. The resulting current is affected by the distance between the pipette tip and sample surface and by the sample surface charge. The feedback keeps this current constant at 99% of the unperturbed current. This leads to a charge dependent tip–sample distance. In situation 1, the sample is negatively charged mica, while in situation 2, it is the cationic lipid DPTAP. The red (blue) line represents the path of the pipette tip at +100 mV (−100 mV) bias potential. The black line represents the path taken had the sample been uncharged. The dashed box indicates the tip–sample region investigated with FEM simulations. The simulation geometry (**b**) is created with rotational symmetry along the pipette axis to reduce the three-dimensional problem to 2D. One electrode, a is positioned at the top of the pipette, the other, b is the outer edge of the water bath. The charged sample, c is positioned at a variable distance *d* from the tip of the pipette. The tip itself is characterized by its inner radius *r*_i_ and an opening angle *θ*. (**c**) The lipids used in the experiments are DPTAP (+1 charge) DPPE (neutral) and DPPG (−1 charge).

A non-zero SCD produces a concentration gradient of counter ions that extends into the solution and creates a measurable change in electrokinetic properties^16^. In this study we investigate the surface conductivity near supported lipid bilayers with a scanning ion-conductance microscope (SICM, illustrated in Fig. 1a), and we demonstrate an imaging mode capable of quantitatively mapping the SCD with nanoscale spatial resolution. The effect of surface conductivity on image formation with SICM has been lacking a thorough analysis. We therefore found it imperative to begin this study with the experimental investigation of differently charged lipids to gauge at the influence of their charge on the image formed by SICM.

Phospholipids found in mammalian membranes are exclusively of zwitter- and anionic character. The main part of the lipids carries a neutral net charge^35^, while multiple negatively charged lipids are important for special functions^5^. Cationic lipids are not native to mammalian cells, but they are of utmost importance in drug delivery applications^8^. Three lipids, an anionic, a zwitterionic and a cationic lipid, were selected to represent the different charge possibilities of lipids, but with minimal change in other properties. The lipids: 1,2-dipalmitoyl-sn-glycero-3-phospho-(1′-rac-glycerol) (DPPG), 1,2-dipalmitoyl-sn-glycero-3-phosphoethanolamine (DPPE) and 1,2-dipalmitoyl-3-trimethylammonium-propane (DPTAP), share a common hydrophobic backbone, and they all have a relatively small hydrophilic headgroup (Fig. 1c). Bilayers formed from the lipids therefore have very similar physical properties, only their intrinsic SCD differs. The bilayers were prepared by the method of spontaneous adsorption and spreading of vesicles^36^. Films of pure lipid were hydrated with the imaging buffer to form bilayer structures, sonicated to break the structures into small unilamellar vesicles, and finally deposited on a mica surface. The lipids have a melting temperature above room temperature, which caused the small vesicles to spontaneously fuse over time^37^. This was used to control the size of adsorbed structures on the mica surface and to create incomplete bilayers or areas with multiple stacked bilayers. The gel phase lipids furthermore displayed a minimal lateral diffusion after adsorption, even on a timescale of several hours.

**Figure 2:**
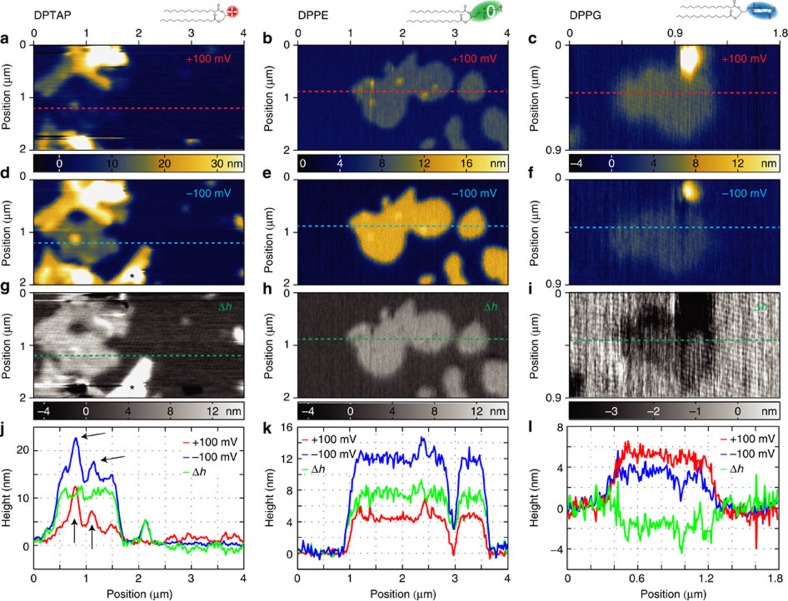
CIHD measurements of phospholipids. SICM images of DPTAP, DPPE and DPPG scanned at bias potentials of first +100 mV (**a**–**c**) and then −100 mV (**d**–**f**). All scans show apparent heights of the lipids that differ from the 5 nm physical height measured by AFM. For DPTAP and DPPE the +100 mV topographies are compressed, while for DPPG it remains somewhat unchanged. At −100 mV the topographies of DPTAP and DPPE are stretched by 8.5 and 7 nm, respectively, while the DPPG topography is compressed by 2.5 nm. (**g**–**i**) The difference Δ*h* between the two apparent topographies; multilayer structures in the lipids vanish giving uniform structures. (**j**–**l**) Line profiles from **a**–**i**. Vesicles on top of the bilayer in **j**, indicated by black arrows, disappear in the Δ*h* profile underlining the topography independence of the CIHD images. During the imaging of DPTAP, a vesicle (*) settled down between scan **a** and **d**, this was excluded from further analysis. DPTAP and DPPE images are 2 × 4 μm^2^ with a pixel size of 20 nm, DPPG is 0.9 × 1.8 μm^2^ (pixel size 8 nm). The colour scales between the ±100 mV images of each lipid are valid for both.

For imaging with the SICM a bias potential of +100 mV was applied to the pipette electrode relative to the bath electrode, and imaging was performed by approaching the pipette to the surface until the current dropped 1% compared with the unperturbed current before the approach, followed by a raster scan of the area of interest. Figure 2a shows typical images of DPTAP structures formed on mica. Flat island structures with stepwise increasing heights were observed; most structures were around 4 nm taller than the substrate, some 9, 14 nm and so on with 5 nm steps. DPTAP samples were also characterized by AFM, showing a height of single bilayers around 5 nm, while two stacked bilayers were 10 nm tall (Supplementary Fig. 1). The height of biological matter measured by AFM and SICM is influenced by surface charges^38^, and while SICM is a non-contact method the force applied in AFM can lead to physical deformation of the sample^18^,^20^. A very low loading force (around 100 pN) was used to minimize deformation, and a height of 5 nm matches the expected height from the molecular structure. A comparison of SICM and AFM images showed that the step height between stacked bilayers was the same (around 5 nm). The measured height of the first DPTAP bilayer was however one nanometre smaller than the physical height when imaged with the SICM at +100 mV bias potential. This height difference is larger than the noise floor (0.5 nm) and is too large to be explained merely by a shift of the lipid surface, when comparing the physical extension of the lipid molecules to the boundary for ion conductance, which is measured by SICM. The height shift was therefore assumed to arise from different surface charge densities of the lipid and the mica substrate.

The influence of surface charge on the SICM signal depends on the applied potential^27^, and while 100 mV is by far the most commonly applied potential, many authors neglect to define their reference electrode (standard convention is to consider the bath electrode as ground). Hence, −100 mV can also be considered a typically used potential. The area in Fig. 2a was scanned again, this time with a bias potential of −100 mV. The resulting image is shown in Fig. 2d. The measured height of the features identified as a single DPTAP bilayer in Fig. 2a is now 13.5±0.8 nm, clearly larger than the physical height of two stacked bilayers. Two and more stacked bilayers also rise up higher than observed when scanning at +100 mV. This shows the clear need for considering surface charges in SICM imaging, as careless data analysis would easily lead to serious misinterpretations. The same procedure of imaging with ±100 mV was applied to samples of DPPE (Fig. 2b,e) and DPPG (Fig. 2c,f), but now the apparent heights of single bilayers were 5.1±0.8 and 12.1±0.8 nm (Fig. 2k) for DPPE and imaging DPPG yielded 3.9±0.8 and 2.5±0.8 nm (Fig. 2l). The noise floor was down to 0.30 nm (Supplementary Note 1). The considerable change in apparent height between the lipids can only arise from the different head groups of the lipids, and thereby the differences in SCD between the lipids.

To separate the contribution of sample topography and effects from the surface charge we subtracted the two topographies obtained at the different potentials from each other. This charge-induced height difference (CIHD, or Δ*h*) image is independent of the sample topography, as any contribution from the physical topography is the same in both images and cancels out in the subtraction. In Fig. 2g–i the topographies measured at +100 mV have been subtracted from the topographies taken at −100 mV. The CIHD images show a uniform Δ*h* for the areas covered with lipids irrespective of the number of bilayers in that area. The line profiles of the DPTAP image (Fig. 2j) nicely visualize this effect; peaks from multilayers sitting on a single bilayer (indicated with black arrows) disappear in the Δ*h* profile. This further supports that the height difference is caused by the difference in SCD between mica and lipid, as the SCD of the lipid is independent of the number of stacked bilayers. The observed Δ*h* is clearly largest for the cationic lipid DPTAP with 9 nm, the value is slightly smaller for the zwitterionic lipid DPPE with 7 nm, and becomes negative with Δ*h*=−2 nm for DPPG. These values clearly indicate the difference in SCD between the three lipids. This procedure can be very useful for any sample type, as it allows the extraction of relative surface charge information (the CIHD images) with the same resolution as standard topological SICM imaging. Extracting absolute SCD values requires a careful theoretical analysis of the ionic flow at the tip apex above the sample.

### PNP analysis

The experimentally observed effect of surface conductivity on SICM imaging can be correlated to the SCD through a theoretical analysis of the nanopipette setup. We perform such analysis by using the coupled PNP equations, a set of formulas widely used to predict and analyse the movement of charged species in chemistry, physics and biology. The ionic current at SICM pipette tips has previously been investigated using the finite-element method (FEM) for solving PNP equations with very good qualitative results^30^,^31^,^32^. These results are however not directly applicable in our case, as the simulations were performed with low-salt concentrations (1–10 mM). The PNP equations are based on a dielectric continuum model, and do not take into account the finite size of ions and correlations between them. The equations will therefore be accurate at the macroscopic scale, but special attention should be shown when applying them to a nanometre-scale geometry. Parallels can here advantageously be drawn to the analysis of nanofluidics, where PNP equations can be used with minor modifications^39^. The nanopipette critical geometry is an order of magnitude larger than the Debye length, and the system is in steady state, which greatly simplifies the required modifications. At physiological salt concentrations the discreteness of ions will play only a very minor role in the bulk solution, but care must still be taken at charged interfaces, where the counter-ion concentration is increased^40^. A general approach relies on modified electrostatic boundary conditions^39^, which will not influence the setup for PNP simulations, but must be accounted for when analysing the result. Here, we present the setup for the FEM analysis of coupled PNP equations, while the special considerations concerning the charged surfaces will be discussed later.

The Poisson equation describes the electrostatic potential *V* in a continuous media of relative permittivity *ɛ* containing the ions *i* of concentration *c*_*i*_ and charge *z*_*i*_:


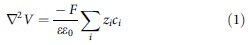


Where *F* is the Faraday constant and *ɛ*_0_ is the vacuum permittivity. Under the assumption that the pipette movement is sufficiently slow convection can be ignored, and the time independent Nernst–Planck equation thus describes the diffusion and migration of the ions:


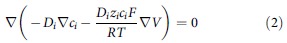


Where *D*_*i*_ is the diffusion constant of ion *i*, *R* is the gas constant and *T* the temperature. The boundary conditions for the NP equation are given as a constant concentration condition or a zero flux condition, and the boundary conditions for the Poisson equation are given as a fixed potential or from the SCD:


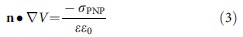


**n** is the surface normal vector and the SCD has been added the subscript PNP due to the aforementioned issues with surface charges in the PNP equations.

Reliable numerical simulations required a mesh size smaller than the Debye length near the charged surfaces, which was only computationally feasible, when using the rotational symmetry of the pipette to reduce the simulation from three- to two-dimensional (2D) (Fig. 1b). Full details of the simulation setup and pipette geometry can be found in Supplementary Fig. 2, Supplementary Tables 1–3 and Supplementary Note 2.

**Figure 3:**
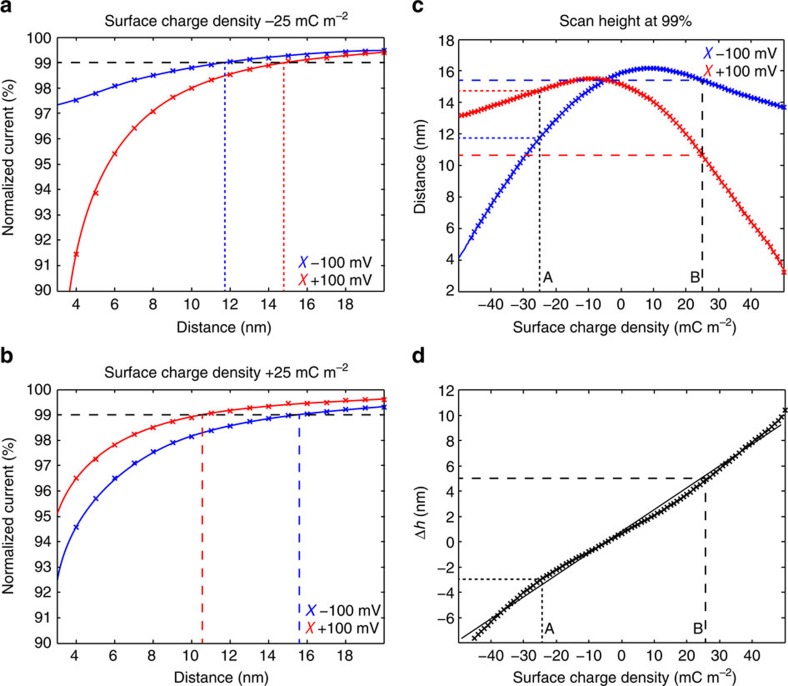
FEM simulations of the QSCM mode. Approach curves from PNP simulations are given to a negatively charged substrate of −25 mC m^−2^ (**a**) and a positively charged substrate of +25 mC m^−2^ (**b**) with bias potentials of 100 mV (red) and −100 mV (blue). The scanning height (99% of unperturbed current) is marked with straight dashed lines in **a** and **b**, and plotted as a function of SCD in **c**. The difference (scanning at negative potential minus positive potential) is shown in **d** and a linear fit is given. The slope (*m*_*h*_=0.172 nm (mC m^−2^)^−1^ or *m*_*σ*_=5.79 (mC m^−2^) nm^−1^) of the linear fit can be used to convert observed changes in scanning height into differences in the SCD.

SICM approach curves were created by simulating the ionic current through the pipette as the distance between tip and sample was reduced in steps of 1 nm. Figure 3a shows approach curves to a surface with a SCD of −25 mC m^−2^, revealing a clear difference between the approach curves from the two applied scanning potentials (±100 mV). The difference between the two curves increases as the distance is decreased, and the 99% mark (usual setpoint for scanning) is reached 3 nm further away from the surface at +100 mV compared with −100 mV. This effect explains a well-known occurrence in SICM imaging with small pipettes, where the pipette is much more likely to ‘crash' at a glass surface when using a negative bias. The approach to a sample with a SCD of +25 mC m^−2^ (Fig. 3b) however shows the opposite effect, the scanning distance is 5 nm higher at −100 mV than at +100 mV. The effect of surface charge on SICM imaging at physiological conditions has previously been ignored with a reference to a Debye length of less than a nanometre^25^, but our results from both experiment and simulation here show that charge differences on a sample can distort the topography with up to several times the Debye length.

The scanning height at 99% unperturbed current as a function of SCD is shown in Fig. 3c, where the scanning height has been obtained from interpolated curves for each SCD from −50 to +50 mC m^−2^ in steps of 1 mC m^−2^. The resulting curves have a maximum close to 0 mC m^−2^, and fall off to the sides with different curvature left and right. The curve of +100 mV is almost a mirror image of the curve at −100 mV, but small differences exist due to the surface charge of the pipette and heterogeneous diffusion constants of the electrolyte. The effect of a given surface charge on the scanning height is visibly different for the two scanning potentials, which explains the different heights obtained for the lipid bilayers in Fig. 2a–f. The shape of the two curves furthermore suggests that two different surface charge densities can give rise to the same distortion of a SICM image; the SCD can therefore not be deduced from a single SICM image even if the physical height of a structure is known. The shift and flip between the two curves gives different distortions, and imaging at two different potentials can therefore resolve ambiguity in the data. The resulting height difference after removing contributions from the topography, as done in the previous section and shown in Fig. 2g–i, can be analysed using these curves. Subtracting the curve for +100 mV from the curve at −100 mV gives the change in scanning height for each SCD (Fig. 3d). Interestingly, this difference between the scanning heights has an almost linear dependence on the SCD with a slope of *m*_*h*_=0.172 nm (mC m^−2^)^−1^ (*r*^2^=0.995). This allows using a simple proportionality factor for the conversion between height difference and SCD. The inverse relation *m*_*σ*_=1/*m*_*h*_ gives the difference in SCD for a measured height difference Δ*σ*_PNP_=Δ*h* × *m*_*σ*_=Δ*h* × 5.79 (mC m^−2^) nm^−1^. The linear fit experiences a maximum error of 0.5 nm or 3 mC m^−2^, which is below the practical resolution presented here, but a more precise model might be needed in future applications with higher resolution.

The precise tip size of nanopipettes will inevitably vary between different experiments, but can be estimated from the measured resistance. The nanopipettes used in the experiments had an inner radius (*r*_i_) around 15 nm and simulations were run with an inner radius of exactly 15 nm. Additional simulations of different pipette sizes (inner radius 10–50 nm) were also performed, to test the influence of *r*_i_. The linear relation between SCD and Δ*h* was conserved with a maximum change in the slope of <10% (Supplementary Figs 3 and 4). The linear relation was also conserved for different scanning setpoints from 97 to 99.5% (Supplementary Fig. 5). Low setpoints (≤98%) could not be used at strongly charged surfaces due to overlap of the potential profiles at very small distances (Supplementary Note 3).

### SCD of lipid bilayers measured by QSCM

**Figure 4:**
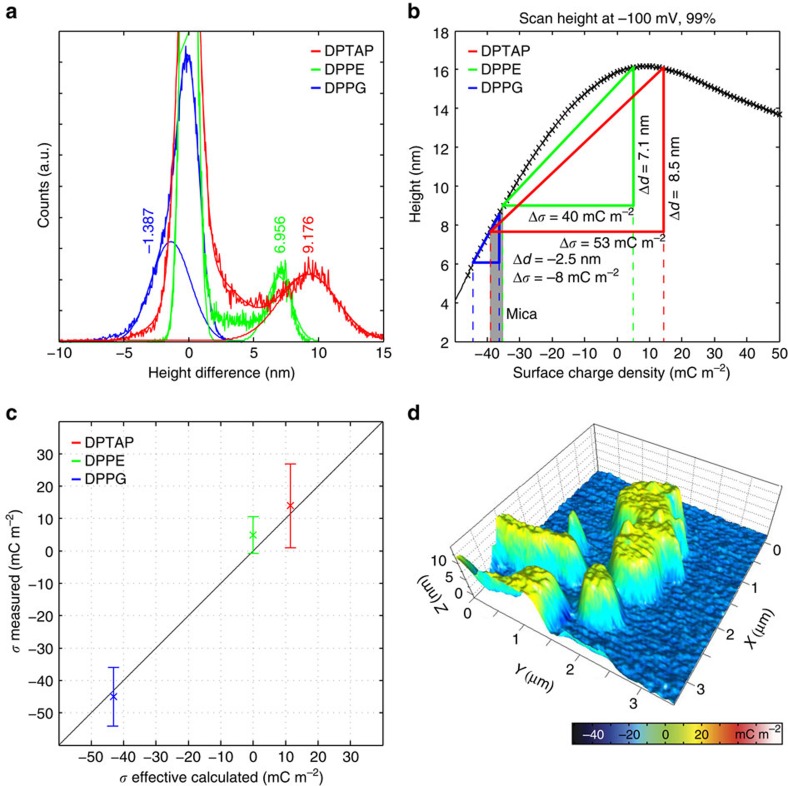
SCD of the three lipids calculated from QSCM measurements. (**a**) Histograms of the Δ*h* images (Fig. 2g–i) of the three lipids with Gaussian distributions fitted to the lipid and mica peaks. The histograms were shifted to centre mica at 0 nm. The lipid peak position times *m*_*σ*_=5.79 (mC m^−2^) nm^−1^ give the difference in SCD between mica and lipid. (**b**) The absolute SCD of lipids and mica is determined by fitting triangles with sides of Δ*d* (height measured at −100 mV subtracted the physical height) and Δ*σ* to the scanning height at −100 mV. The values of mica from the three measurements match within errors. (**c**) Measured SCD versus theoretical SCD for the three lipids. For all the three lipids, experimental and theoretical values match within errors (s.d.). The black line is 1:1 to guide the eye. (**d**) Overlay of DPPE topography and charge map. Topography is taken from Fig. 2e, while the charge is calculated as Δ*h* times *m*_*σ*_=5.79 (mC m^−2^) nm^−1^. Charge and topography co-localize extremely well.

The surface charge densities of the lipid bilayers can now be obtained by applying the PNP analysis to the SICM images in Fig. 2. The relative SCD is calculated using the proportionality factor obtained in the previous section: Δ*σ*_PNP_=Δ*h* × *m*_*σ*_=Δ*h* × 5.79 (mC m^−2^) nm^−1^, where Δ*h* refers to a measured height difference, when comparing scans at +100 and −100 mV. This formula has general validity, it can be applied to two of the main SICM imaging modes, DC^17^ and hopping mode^41^,^42^,^43^ or it could be combined with a new imaging scheme with variable bias potential during scanning. For each of the three lipids imaged in Fig. 2 the difference in SCD (Δ*σ*_PNP_) is calculated using histograms of the CIHD images (Fig. 4a; Supplementary Fig. 6 with fitting parameters in Supplementary Table 6). The histograms of DPTAP and DPPE have two distinct peaks, which can be attributed to the mica substrate and lipid structures, respectively. Gaussian distributions have been fitted to the histograms and the distance between the peaks, Δ*h*, is used to calculate Δ*σ*_PNP_. The width of the peaks is used to estimate the standard deviation (s.d.) of the measurement. For DPTAP, a Δ*h* value of 9.2 nm is obtained corresponding to Δ*σ*_PNP_=53.1 mC m^−2^ (±12.8 mC m^−2^, s.d.) and for DPPE Δ*h* is 7 nm corresponding to Δσ_PNP_=40±5.6 mC m^−2^. The existence of two peaks is not immediately obvious in the histogram of the DPPG sample, but the histogram is reproduced nicely with the sum of two Gaussians, while a single Gaussian is clearly not enough (Supplementary Fig. 6c). The resulting Δ*h* of −1.4 nm corresponds to Δ*σ*_PNP_=−8±9.0 mC m^−2^ for DPPG.

The relative SCD can provide important information about lipid systems, but quantitative values are required for identifying the true electrostatic properties. To correlate the relative SCD difference of lipid compared with the mica to the absolute SCD, additional information about the system is required; here we use the physical height of the lipid bilayer. This can be obtained for example with AFM measurements. For the lipids in this study a height of 5 nm was assumed for a single bilayer. The distortion of scanning height, Δ*d*, at one of the bias potentials can be determined, as this is the apparent height of a bilayer minus its physical height. Going back to the calculated scanning height—SCD curve, (Fig. 3c) a right triangle with sides' Δ*σ* (horizontal) and Δ*d* (vertical) can be drawn into one of the curves such that two vertices will coincide with the curve. The lateral positions of the two vertices correspond to the SCD of mica and lipid, respectively. For all the three samples a unique position satisfying this condition could be found, as demonstrated for the scans at −100 mV in Fig. 4b. The left vertex corresponds to mica for the DPTAP and DPPE scans, while the right vertex of the DPPG scan corresponds to mica due to the negative scan height distortion. The SCDs assigned to mica by this method were −38±4.7, −36±3.1 and −35±4.7 mC m^−2^, where the difference between the individual values is less than the s.d. (±4.2 mC m^−2^) . With this, we obtain an average SCD of *σ*_mica_=−36.3±4.2 mC m^−2^. For the lipids, we obtain the following values: *σ*_DPTAP_=15.1±12.8 mC m^−2^, *σ*_DPPE_=5.3±5.6 mC m^−2^ and Δ*σ*_DPPG_=−44.0±9 mC m^−2^. The SCD of mica and lipids are obtained by applying the PNP analysis to experimental data and the values follow the trend that the positively charged lipid has a positive charge, the zwitterionic is close to zero, while the negatively charged lipid has a negative SCD.

### Single-point surface charge measurement

The characterization of lipid bilayers on mica is based on the stepwise height difference between mica and lipid or between two stacked lipid bilayers. QSCM can however also be applied to a homogeneous surface through the linear correlation between Δ*h* and SCD. Approach curves to SiO_2_, mica and mica modified with 3-(aminopropyl)triethoxysilane (APTES) are given in Supplementary Fig. 7; with a detailed description of the results in Supplementary Note 3. The change in distance to the sample at a 99% setpoint, Δ*h*_99%_, was measured and converted to SCD with values for mica of −5±1 nm translating to −33.6±5.8 mC m^−2^. The uncertainty related to this value is a rough estimate as errors related to drift are problematic to quantify and will be discussed later. The measured surface charge densities of the remaining substrates, 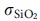
=−16.3±5.8 mC m^−2^ and *σ*_APTES_=1.1±5.8 mC m^−2^, matched expectations (detailed description in Supplementary Note 3).

## Discussion

The new mode, QSCM, measures the surface conductivity arising from a specific SCD. The SCD is calculated on the basis of the PNP equation, and the assumption that the surface conductivity is created solely by an enrichment of counter ions near the surface. Per definition the surface conductivity is the additional conductivity of an electrolyte solution near a charged surface, and the additional conductivity can come from conduction directly on the surface, conduction in the stern layer or conduction within the remaining electrical double layer^44^. The lipid bilayer consists of separated molecules and conduction directly on the surface can be neglected. The ion concentration in the electrical double layer is considerably higher than in the bulk, and PNP equations would not be appropriate for describing the ionic transport here^40^. The SCD boundary values applied in the PNP analysis are therefore not strictly the physical SCDs, but they represent an effective SCD that would be obtained when assuming the Poisson–Boltzmann equation to preserve from the bulk to the surface^34^. Practically all electrokinetic experiments use this assumption, and the SCD obtained by QSCM can be compared with the results obtained with other methods. A precise description of the effective SCD and of the Gouy–Chapman–Grahame–Stern model^33^ and the extended Poisson–Boltzmann approximation^34^ is given in Supplementary Note 4. Using these formulas the effective SCD was calculated from the physical characteristics of each lipid (Table 1).

A precise comparison to the theoretically calculated values shows that the measured values for DPTAP and DPPG match within error, while DPPE shows a small unpredicted positive charge (Table 1; Fig. 4c). This deviation for DPPE comes from an oversimplification in the theoretical model, as it assumes that all charges are located at the physical boundary of the lipid bilayer, while the conformation of charged groups in zwitterionic lipids can give rise to an effective surface charge^45^. The arrangement of the headgroup in DPPE has been shown to be influenced by the ionic strength of the electrolyte in a way that the SCD at physiological conditions is expected to be slightly positive^46^. In comparison, the charge in phosphoglycerol (PG)-lipids is believed to be close to the bilayer surface^47^, and the same is to be expected for DPTAP due to the small size of the hydrophilic headgroup. The SCD of mica was measured to −36.3±4.2 mC m^−2^ from the lipid scans and −33.6±5.8 mC m^−2^ using a single-point charge measurements. A theoretical calculation of the SCD for mica is complicated due to adsorption of electrolytes to the crystal structure, but a surface complexation model used by Mugele *et al*.^48^ gave a value of −47 mC m^−2^ (estimate from graph) at 150 mM NaCl and pH 6 corresponding to an effective SCD of −42 mC m^−2^ with the extended Poisson–Boltzmann approximation. The negative SCD of mica increases slightly with increasing pH, which would predict a stronger charge on the surface than measured here. Experimental measurements of the zeta-potential using streaming potential predicted a SCD of −63 mC m^−2^ (calculated using the Grahame equation)^49^, but the zeta-potential is not directly measurable^50^, and literature values vary depending on the method. Comparison of the measured SCD to literature zeta-potential values require careful consideration, as the zeta-potential is often used solely as a quality-control tool for comparison of samples^50^. The zeta-potential of lipid bilayers is measured using a volume of small liposomes, where the curvature of the structures and a different surface area per lipid could significantly change the zeta-potential compared with a flat bilayer. QSCM on the other hand measures the SCD of a small area and can be used as a quality-control tool, but also for determining the true SCD.

The SCD of lipid bilayers was mapped using QSCM with a precision of up to 5.6 mC m^−2^. The charge values matched accurately with theoretically calculated values (Fig. 4c), successfully demonstrating SCD mapping at a physiological salt concentration. The SCD precision is limited by instrumentation, noise and edge effects of the bilayer structures. Thermal noise (Supplementary Note 5) provided the main limitation in the characterization of DPPE bilayers (s.d. 5.6 mC m^−2^) and mica (s.d. 4.2 mC m^−2^), while multiple bilayers and attached vesicles created a larger standard deviation for DPTAP bilayers (s.d. 12.8 mC m^−2^) and DPPG (s.d. 9 mC m^−2^). The precision could have been improved by the use of a lower amplifier bandwidth (possibly requiring a slower scan speed) or by smoothing and frequency filtering of the data, but this would be at the expense of longer imaging time and lower spatial resolution. The pipette was moved in a lateral direction, and drift would only have a minimal influence on SCD resolution as Δ*d* was measured on a very short timescale. This was however not the case in the single-point charge measurement, where the pipette was moved in a large vertical motion. The vertical speed of the pipette (100 nm s^−1^) created a gap of several seconds between each measurement, which made the value of Δ*h*_99%_ vulnerable to vertical drift. A higher pipette velocity would reduce the time gap, but could also lead to delays in the ion current due to ion diffusion limitations^51^. Multiple approach curves could increase the practical resolution of the single-point measurement, but this approach should be used with great care for vertical drift, which often occurs in practical systems.

The QSCM mode is almost independent of the nanopipette geometry as revealed in Supplementary Figs 3 and 4. The ionic current in SICM depends on the pipette size, and the scanning height scales proportionally with the pipette radius (when neglecting surface charges). A measurement of SCD based directly on the measured current would therefore require a precise knowledge of the pipette size and geometry. In contrast, the QSCM mode uses the CIHD, which is independent of pipette size, tip angle and SCD of the pipette walls. This, combined with a spatial resolution equal to that of DC-mode SICM, makes the QSCM mode both a useful and robust analysis tool for future studies.

The high-spatial resolution of SICM makes it capable of imaging single proteins in a membrane^22^, and QSCM could likely be employed to investigate the interplay between charged membranes and proteins. Live-cell imaging is also a possibility, although this will require attention to possible intrinsic interactions, local slope of the surface and other imaging parameters^27^,^52^. The use of higher setpoints (>99%) might be required for imaging live cells^41^, and simulations show that QSCM can be used at 97–99.5% (Supplementary Fig. 5). However, the effect of thermal noise increases with setpoint (Supplementary Note 5), and the SCD precision will decrease at >99%. QSCM could alternatively be applied to more static membranes to investigate the charge of lipid rafts, or be applied to investigate DNA–lipid interactions to optimize the charge composition for transfection vesicles^7^.

The use of QSCM spans widely; it is not limited to lipid bilayers, and the high-quantitative precision could provide a new approach for characterizing organic as well as inorganic surfaces. The approach of using a nanopipette, combined with an extensive theoretical analysis, will in the future provide valuable structural and functional information for biological samples.

## Methods

### SICM imaging

All SICM images were recorded using an XE-Bio system (Park Systems, Suwon, South Korea). The instrumentation has been described previously^23^. The setup uses a Femto DLPCA 200 amplifier and a Nikon Eclipse TI-U optical microscope.

The imaging buffer consisted of 150 mM NaCl (Sigma, USA) and 10mM HEPES (Sigma, USA) in milli-Q grade water adjusted to pH 7. During imaging the temperature was kept at 20 °C. The SICM was operated in constant current mode (DC). Before each scan the unperturbed current far from the surface was measured and a 1%-drop setpoint calculated. This low setpoint (99%) was chosen to optimize the signal to noise ratio, while higher setpoints (99.5–99.7%) are often used for live-cell imaging to prevent tip–sample collisions^41^. After the measurement the unperturbed current was measured again. Scans with a drift in unperturbed current of more than 0.3% were discarded. Images were scanned subsequently with bias potentials of +100 and −100 mV. A period of 2–5 min was introduced following a change of the potential to assure a stable current.

### Single-point surface charge measurement

Current-distance curves were recorded in rapid succession to minimize drift between each approach. The pipette was moved vertically at a speed of 100 nm s^−1^ towards the surface until a reduction in current of 3% was measured, at which point the pipette was retracted at a speed of 100 nm s^−1^. This procedure was repeated 10 times; 5 times with a pipette potential of +100 mV and 5 times with a pipette potential of −100mV. Substrates were prepared immediately before used: mica was cleaved using adhesive tape and submersed in the imaging buffer for 30 min before imaging. A silicon wafer with a 200 nm top layer of SiO_2_ was cleaned by sonication in acetone, rinsed with ethanol and N_2_ blow dried before imaging. A freshly cleaved mica plate was coated with APTES (Sigma, USA) by vapour exposure for 1 h using a 2 l desiccator clock and 100 μl APTES solution.

### Nanopipettes

Nanopipettes were prepared using a CO_2_-laser-based micropipette puller (P-2000, Sutter Instruments, Novato, CA, USA). Ten centimetre fire-polished borosilicate filamented capillaries of 0.50/1 mm inner/outer diameter (Sutter Instruments, USA) were pulled using the following parameters: Heat 310, Fil 4, Vel 25, Del 225, Pul 150, resulting in pipette tips of 14–20 nm inner radius and an outer half-cone angle of 3°, the angle was measured using an optical microscope, and the radius was calculated from a measured resistance of 300–450 MΩ of the pipettes before imaging.

### Supported lipid bilayers

DPTAP, DPPE and DPPG were obtained in organic solutions from Avanti Polar Lipids (USA). Aliquots of lipid solution were placed in glass containers in a vacuum desiccator overnight forming dry lipid films. The lipid films were then resuspended in the imaging buffer to a final concentration of 1 mg ml^−1^, and sonicated in a bath sonicator for 30 min at room temperature. Overall, 5 μl vesicle solution was added to a 1 cm^2^ freshly cleaved mica plate and allowed to settle to form an incomplete bilayer. The plate was then gently rinsed with 5 ml imaging buffer to wash off excess lipids and stored in buffer until further use.

### AFM imaging

Lipid samples on mica were characterized by PeakForce Tapping mode AFM using a Dimension FastScan (Bruker, USA). All scans were performed in the imaging buffer using ultrasharp silicon tip cantilevers (FastScan-C, Bruker, USA) with a 0.8 N m^−1^ force constant and 5 nm tip radius.

### Image processing and calculation of SCD

Image processing was done with SPIP (Image Metrology, Hørsholm, Denmark). The topography images received a line wise offset and global plane correction, no smoothing filters were applied. Special care was taken to obtain images with a background as flat as possible. Images were aligned using the built in alignment function. The topography with positive bias potential was then subtracted from the topography with negative bias potential. The resulting height difference image was converted into the SCD map using a conversion factor of *m*_*σ*_=5.79 (mC m^−2^) nm^−1^. The average value of the SCD difference between mica and the lipid was obtained via the histogram of the height difference image. The sum of two Gaussian peaks was fitted to the histogram to determine the centre position of the two peaks; the obtained fit coefficients are listed in Supplementary Table 6. Absolute values of the SCD were obtained by fitting the scan height distortion (Δ*d*) at −100mV and the relative SCD (Δ*σ*) to Fig. 3c. Alternatively, the absolute SCD of mica could be used in combination with Δ*σ*, which produced the same results. The histograms provide information about the uncertainty of the measurement as well. The uncertainty in SCD is equal to the uncertainty in the height times the conversion factor *m*_*σ*_ , hence the standard deviation can be calculated from the width of the Gaussian fit to the corresponding peak (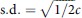).

### Finite-element analysis of ion-conductance

The ionic current behaviour was investigated using a FEM for solving Poisson (P) and Nernst–Planck equations. A 2D triangular mesh was created to mimic the rotational symmetry of the SICM setup, and PNP equations were fully coupled and solved using boundary conditions matching the expected experimental conditions. Standard conditions for simulations consisted of a pipette with inner tip radius of 15 nm, outer/inner radius ratio of 2, half-cone outer angle of 3°, pipette SCD of −25 mC m^−2^, pipette bias potentials of ±100 mV and sample surface charge densities between −50 and 50 mC m^−2^. Simulations were performed using COMSOL Multiphysics v4.4, LiveLink and MATLAB. Full details of the simulation geometry, variables and constants can be found in Supplementary Fig. 2 and Supplementary Tables 1–3.

### Data availability

Data supporting the findings of this study are available within the article (and its Supplementary Information files) and from the corresponding author upon reasonable request.

## Additional information

**How to cite this article**: Klausen, L. H. *et al*. Mapping surface charge density of lipid bilayers by quantitative surface conductivity microscopy. *Nat. Commun.* 7:12447 doi: 10.1038/ncomms12447 (2016).

## Supplementary Material

Supplementary Information Supplementary Figures 1-7, Supplementary Tables 1-6, Supplementary Notes 1-5 and Supplementary References.

## Figures and Tables

**Table 1:** Measured and calculated values for the lipids used in the study.

	**Gel transition temp (°C)**	**Surface area (Å^2^)**	**pKa**	***σ*** (mC m^−2^)*	***σ***_**eff**_ **(mC m^−2^)**†	***σ***_**PNP**_ **(mC m^−2^)**‡
DPPE	63 (ref. 53)	39.9 (ref. 54)	11.1 (Nh3^+^) (ref. 55) ≤1 (P04^−^) (ref. 55)§	0	0	5.3±5.6
DPPG	41 (ref. 53)	46.7 (ref. 56)	2.9–3.1 (P04^−^) (ref. 55)	−188	−42.8	−44±9
DPTAP	45 (ref. 57)	46.0 (ref. 58)	—‖	348	11.5	15.1±12.8
Mica						−36.3±4.2

The lipids were all expected to be in gel phase at room temperature, and literature values for surface area and dissociation constants are, as far as possible, matching the experimentally used conditions.

^*^Surface charge density calculated from the Gouy–Chapman–Helmholtz–Stern model.

^†^Effective surface charge density calculated from the EPB approximation.

^‡^Surface charge of lipid bilayers measured by QSCM.

^§^values for (12:0)_2_ PE.

^‖^The trimethylammonium group in DPTAP is a quaternary ammonium and is expected to be charged at all pH values.
