# Supplementary material for: Mapping surface charge density of lipid bilayers by quantitative surface conductivity microscopy
Source: Nat Commun. 2016 Aug 26;7:12447. doi: 10.1038/ncomms12447 (PMC5007656; doi:10.1038/ncomms12447)
Supplement: Supplementary Information — Supplementary Figures 1-7, Supplementary Tables 1-6, Supplementary Notes 1-5 and Supplementary References. [file ncomms12447-s1.pdf]

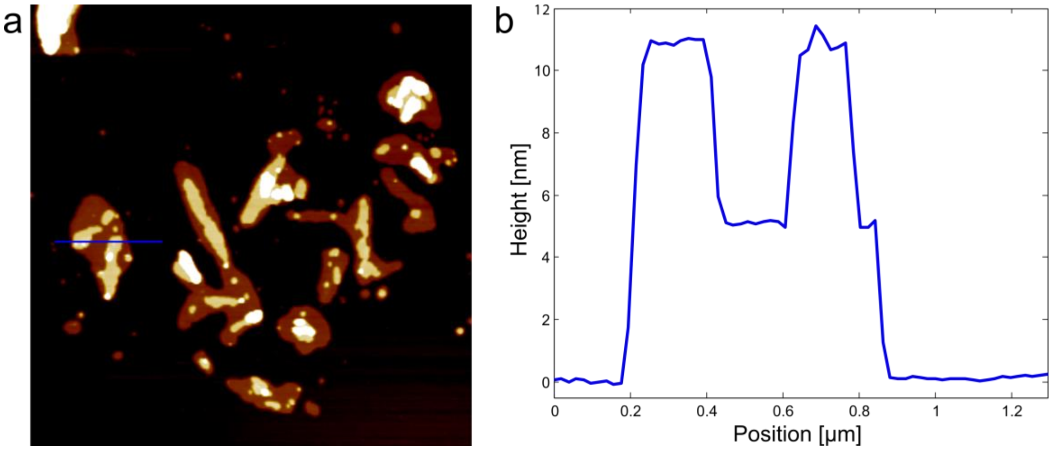

Supplementary Figure 1. AFM scan of DPTAP bilayers. (**a**) Topography image with distinct single, double and triple layer structures. (**b**) Line profile corresponding to the line in (a) of a single bilayer structure (5 nm) and double bilayer structures (10-11 nm). Imaging was performed using tapping mode in water with 150 mM NaCl and 10 mM HEPES at pH 7.0.

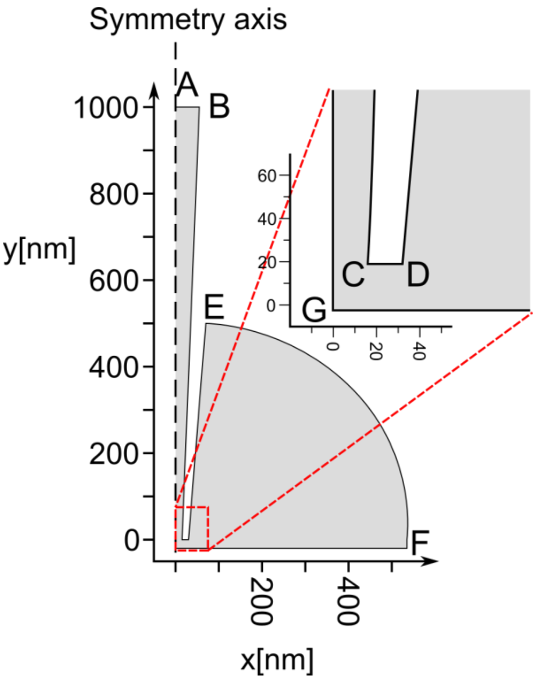

Supplementary Figure 2. Nanopipette geometry used for FEM simulations. The pipette-sample geometry was mimicked using the 2D rotational symmetry around the pipette centre line. The grey area is the solution filled pipette and bath. Boundaries between letters (A to G) are described in Supplementary Table 1. Figure to scale.

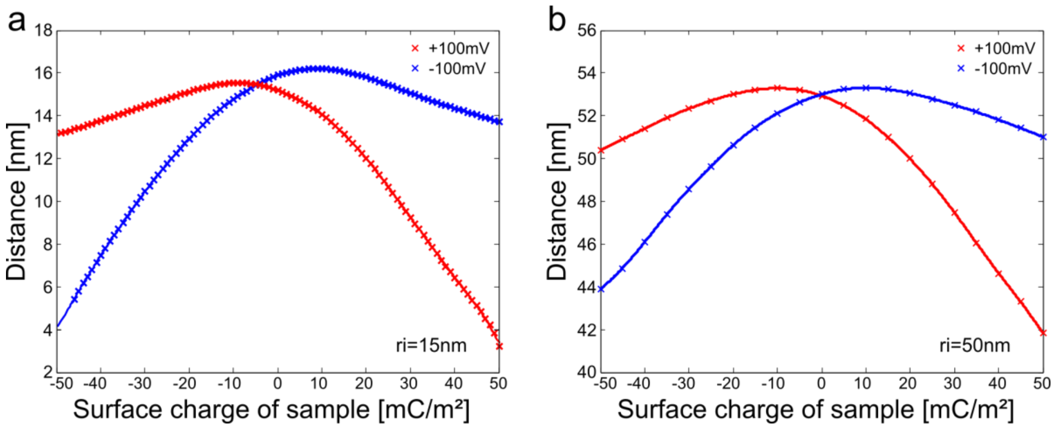

Supplementary Figure 3. Scan height dependence on pipette size. (**a**) The scanning height for a pipette of 15 nm inner radius at a 99% setpoint. (**b**) 50 nm inner radius pipette. The scanning height over a neutral surface is approximately equal to the inner radius, while the absolute change in scanning height due to surface charge is similar for the two pipettes.

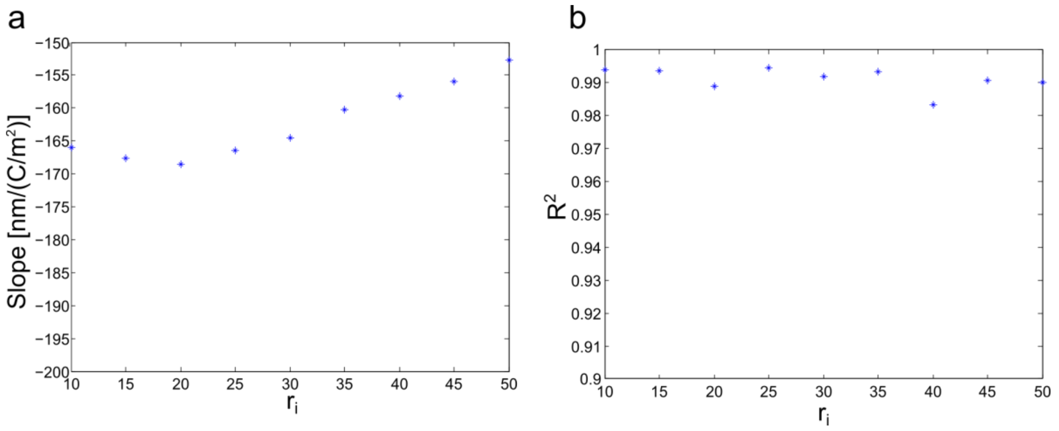

Supplementary Figure 4. Linear correlation between Δ*h* and SCD in simulations with different pipette sizes. (**a**) The slopes of linear fits to Δ*h* as a function of surface charge density obtained for different inner radii of pipettes in PNP simulations. (**b**) R^2 values of the linear fits. The inner radius of the pipette was increased from 10 to 50 nm in the simulation geometry, while the pipette outer radius was set to double the inner radius. The linear correlation is preserved as seen from R^2 values, while the slope value is almost unchanged. The pipette and bath sizes were increased for the simulations and fewer values of SCD included, giving a small change in the slope value compared to results presented in the main text.

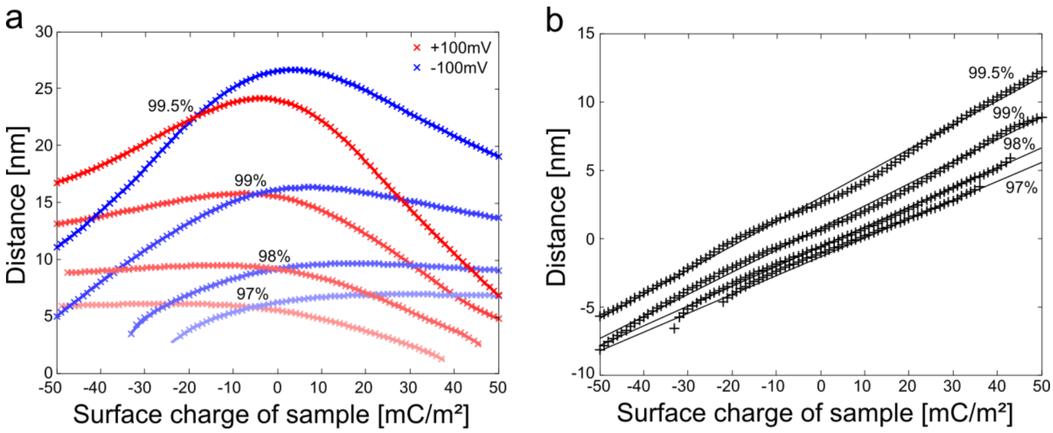

Supplementary Figure 5. Linear correlation between Δ*h* and SCD at different setpoints. (**a**) The scanning height at 97%, 98%, 99% and 99.5% setpoints as a function of surface charge density. The plot is similar to Figure 3c. (**b**) The difference in height (scanning at a negative potential minus positive potential) with linear fits. The plot is similar to Figure 3d.

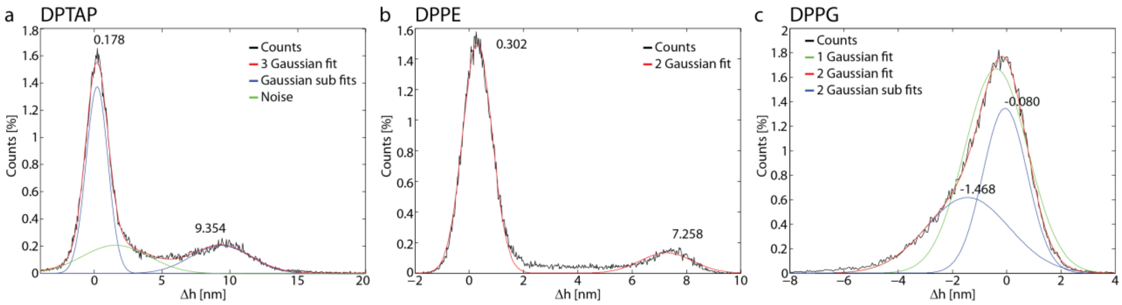

Supplementary Figure 6. Height distribution of the charge induced height difference (CIHD) maps (Fig. 2g-i) with fitted Gaussian distributions. The black lines shows raw data, while red lines are the sum of Gaussian fits used for data analysis. The blue lines denote the peaks for mica and lipid respectively. (**a**) DPTAP; the inclusion of a third Gaussian peak attributed to noise or edge effects was necessary to obtain a good fit. (**b**) DPPE; peaks were well separated and individual curves would coincide with the sum. (**c**) DPPG; the two Gaussian distributions overlap, but a single Gaussian fit (green) clearly provides a much poorer match than the sum of the Gaussians. The fitting parameters for all curves are listed in Supplementary Table 6.

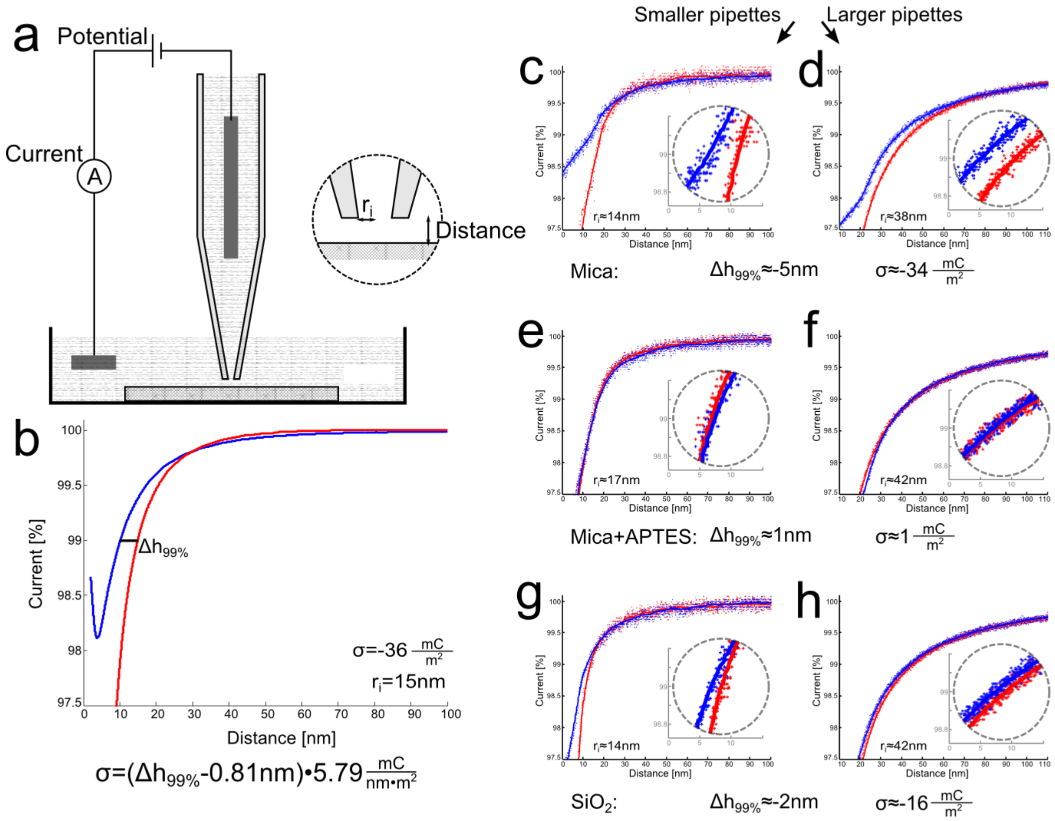

Supplementary Figure 7. Single point charge measurements: The surface charge density of different materials experimentally evaluated at single points using the topography correction scheme of Figure 3. (**a**) Schematic of method. (**b**) Simulated approach curve. (**c,d**) Approach curves to mica using a small pipette (**c**) and a large pipette (**d**) showing a significant difference between curves from different potentials. (**e,f**) Approach curves to mica coated with APTES using a small pipette (**e**) and a large pipette (**f**) showing only a minor difference between curves from different potentials. (**g,h**) Approach curves to SiO_2 using a small pipette (**g**) and a large pipette (**h**) showing a clear difference between curves from different potentials. The inner tip radius, r_i, of the pipette was estimated from the measured current.

Boundary	Description	Ion flow (NP)	Potential (P)
AB	Pipette top / electrode	Reservoir: $c_i=c$	Electrode: $V=V_0$
BC	Pipette inner wall	Isolating: $n\bullet N_i=0$	Surface charge: $\nabla V=-\sigma_p/\varepsilon_0\varepsilon$
CD	Pipette tip	Isolating: $n\bullet N_i=0$	Surface charge: $\nabla V=-\sigma_p/\varepsilon_0\varepsilon$
DE	Pipette outer wall	Isolating: $n\bullet N_i=0$	Surface charge: $\nabla V=-\sigma_p/\varepsilon_0\varepsilon$
EF	Water bath / electrode	Reservoir: $c_i=c$	Electrode: $V=0$
FG	Sample surface	Isolating: $n\bullet N_i=0$	Surface charge: $\nabla V=-\sigma_s/\varepsilon_0\varepsilon$
GA	Symmetry axis	Symmetry	Symmetry

Supplementary Table 1. Boundary conditions for simulation setup. The boundaries in the simulation setup, shown in Supplementary Figure 2, are defined according to the physical pipette-sample geometry. Boundary conditions for ion flow in the Nernst-Planck equation and potential in the Poisson equation are given as absolute or differential.

Parameter	Unit	Standard	Additional tested values
σ_s	mC m^{-2}	-50:1:50	-50:5:50
r_i ($r_o=2r_i$)	nm	15	10:5:50
r_o (r_i=15nm)	nm	30	20:5:60
σ_p	mC m^{-2}	-25	-0.05:0.01:0
Θ	°	3	2:0.5:7

Supplementary Table 2. Pipette geometries tested in simulations. Different pipette geometries were used for PNP simulations in order to test the robustness of the QSCM method. Variations in the inner radius (r_i), ratio of outer to inner radius (r_o), surface charge density of the pipette material and pipette outer half angle all had minor effect on the linear correlation between Δh and sample SCD.

Constant	Unit	Value
c	mol m^{-3}	150
D_+	m^2 s^{-1}	$1.33*10^{-9}$
D_-	m^2 s^{-1}	$2.03*10^{-9}$
ε		78
T	K	297

Supplementary Table 3. Simulation constants. The concentration (c), relative permittivity (ε) and temperature (T) used in simulations. D_+ is the applied diffusion constant of sodium ions and D_- is the applied diffusion constant of chloride ions[1].

Relative permittivity of the Stern layer ε_s	Calculated effective surface charge σ_{eff_nl} of DPPG [mC m^{-2}]
78	-42.8
60	-48.0
40	-54.8
20	-57.9

Supplementary Table 4: Effect of Stern layer capacitance. The Stern layer capacitance affects the calculated effective surface charge density of the DPPG bilayer. A value corresponding to the bulk solution was used for results presented in the main text, but a lower relative permittivity would not affect the calculated value significantly.

Lipid	σ_{eff_lpb} [mC m^{-2}]	σ_{eff_nl} [mC m^{-2}]
DPPG	-36.0	-42.8
DPPE	0	0
DPTAP	11.3	11.5

Supplementary Table 5: Calculated effective surface charge density of the three lipids. The effective surface charge density of the three lipids was calculated using the Gouy-Chapman-Grahame-Stern model and the extended Poisson-Boltzmann approximation. The linear Poisson-Boltzmann approximation (lpb) is often used, but this becomes imprecise at very high/low surface charge densities. Results from the non-linear Poisson-Boltzmann model (nl) were used in the main text.

	a1	b1	c1	a2	b2	c2	SSE	RMSE
DPPG	1.359 (1.346, 1.372)	-0.08031 (-0.08302, -0.07761)	1.16 (1.153, 1.166)	0.5994 (0.591, 0.6078)	-1.468 (-1.497, -1.439)	2.194 (2.179, 2.21)	0.01308	0.005146
DPPE	1.505 (1.491, 1.519)	0.302 (0.2963, 0.3077)	0.7667 (0.7586, 0.7748)	0.1327 (0.1224, 0.143)	7.258 (7.172, 7.345)	1.367 (1.244, 1.49)	0.6119	0.0352
DPTAP	1.374 (1.372, 1.376)	0.1775 (0.1764, 0.1785)	1.141 (1.139, 1.143)	0.2069 (0.2062, 0.2077)	9.354 (9.34, 9.367)	3.118 (3.099, 3.136)	0.0104	0.002641

Supplementary Table 6: Fit coefficients for the Gaussian fits in Figure 4a and Supplementary Figure 6. Fits were performed in MATLAB using the following library model: General model Gauss2: f(x) = a1*exp(-((x-b1)/c1)^2) + a2*exp(-((x-b2)/c2)^2). Coefficients are in nanometres and given with 95% confidence bounds. The first peak is the peak associated with the mica background, the second peak is for the Lipid. For the DPTAP a third Gaussian attributed to random background noise was needed to obtain a good fit. Its coefficients are: a3 = 0.209 (0.2071, 0.211); b3 = 1.557 (1.534, 1.58); c3 = 3.497 (3.472, 3.522).

Supplementary Note 1

Experimental resolution of QSCM. The resolution of measured surface charge density is given by the vertical resolution of SICM scans. A precise determination of vertical resolution is however problematic for a scanning probe microscope, as it is influenced by imaging parameters such as gain, integration times and scanning speed. What is easier to measure, and conveys important information, is the noise floor of the measurement, any signal smaller than this will be lost in the noise. In QSCM measurements the noise floor can be determined by considering the apparent surface roughness of bare mica. Mica should be atomically flat and exhibit a uniform surface charge density (the periodicity of the crystal structure is much smaller than the pipette size). For the DPPE sample shown in Fig. 2 b and e the measured surface roughness (1 SD) of mica is 0.30 nm for the +100 mV scan and 0.35 nm for the -100 mV scan (slightly larger than the thermal noise of 0.28 nm and 0.19 nm). This shows an achieved sub-nanometre vertical resolution with the used imaging parameters. In the CIHD image, after subtraction of two images, the roughness is 0.55 nm. The resulting error, when adding two measurements with independent Gaussian noise, is given by convolution of the two Gaussians. Hence $\sigma_{sum} = \sqrt{\sigma_1^2 + \sigma_2^2}$, which in this case gives a theoretical minimum of 0.46 nm. That the theoretical minimum is not obtained could be due to a remaining large scale tilt in the images or the effect of unfiltered non-Gaussian noise like line noise.

The roughness in the CIHD image corresponds to a noise floor of 3.2 mC m^{-2} when measuring the SCD. The conversion from CIHD images (in nm) to SCD images (in mC m^{-2}) is based on a conversion factor obtained from FEM-simulations. The approximation of this conversion factor as a constant might introduce an error with a maximum of 0.5 nm or 2.9 mC m^{-2}. The error could be eliminated by the use of a higher order fit to the FEM results, or the use of a lookup table, but this would complicate the data analysis correspondingly.

Supplementary Note 2

Poisson-Nernst-Planck Simulation Setup. The ionic current behaviour at the nanopipette tip in the SICM setup was simulated using a finite element method for solving Poisson and Nernst-Planck equations. The simulation setup includes only the area around the tip of the pipette, while the distance from tip to pipette electrode is approximately 1 cm and the distance from tip to batch electrode around 2 cm. This simplification is justified by the neglectable resistance of the large pipette away from the tip and the bulk electrolyte solution between pipette and bath electrode. The simulation geometry is shown to scale in Supplementary Figure 2. In this setup the ratio between inner radius and wall thickness is kept constant from pipette tip to top. The geometry boundaries are described in Supplementary Table 1 with boundary conditions for both Poisson and Nernst-Planck equations.

The ionic current was measured as the integral over the total charge passing a boundary spanning the inside of the nanopipette:

$$I = \int_0^A F \sum_i z_i N_i \, dl \qquad (1)$$

Where N_i is the movement of ion i over the boundary and F is the Faraday constant.

The pipette geometry in Supplementary Figure 2 corresponds to the pipettes used in experiments for characterizing lipid bilayers. The precise shape of the pipette tip is not known due to the limitations of characterization techniques; optical microscopy can only estimate the outer angle due to the limited resolution and SEM requires a conductive coating, which will change the geometry. Different authors report different shapes of pipettes that were otherwise produced in the same way, especially the ratio between outer radius (r_o) and inner radius (r_i) at the tip is disputed. Traditionally the ratio is assumed to remain constant from bulk to tip[2], but recent studies have reported both increasing and decreasing wall thickness[3–6]. An outer/inner ratio of 2 (corresponding to bulk filament) was assumed for simulations presented in the main text, but ratios from 1.3 to 4 were tested and showed only minor effects on the Δh to SCD relation. The linear correlation characteristics between Δh and SCD from simulations with different values for the size of the pipette (r_i when r_o=2r_i) is shown in Supplementary Figure 3 and 4. The pipette length was increased to 3 μm and the bath radius to 1.5 μm. Simulations with different outer half cone angle (Θ) and surface charge density of the pipette walls (σ_p) were also performed, but again showed

no significant changes in the linear relation of Δh to SCD. A full list of the used variables is given in Supplementary Table 2 and relevant simulations constants are listed in Supplementary Table 3.

Supplementary note 3

Single point surface charge density measurement. The surface charge density of different materials was evaluated using QSCM by an investigation of current-distance curves. Supplementary Figure 7b shows a simulated current-distance curve to a surface of -36 mC m^{-2}, which was the surface charge density measured for mica using the mapping approach. The curves at pipette potentials of +100mV (red) and -100mV (blue) clearly differ due to the highly charged surface. The difference in height at 99% current ($\Delta h_{99\%}$) is approximately -5 nm, this corresponds within error to the linear correlation between surface charge density and height difference described in Figure 3d:

$$\sigma = (\Delta h_{99\%} - 0.81\mathrm{nm})5.79\,\frac{\mathrm{mC}}{\mathrm{nm}\cdot\mathrm{m}^2} \qquad (16)$$

The current-distance curve at -100 mV shows an odd behaviour at below 3 nm, where the current suddenly increases as the distance is reduced. This behaviour is related to an overlap of potential profiles from the pipette and sample surface, where the surface charge density is fixed, but the surface potential can increase exponentially as it is reflected between the surfaces. Simulations using a constant potential[14] boundary condition were tested, but they showed only minor effects on the current at distances above 3 nm and would therefore not influence the remaining results.

If a pipette is brought into contact with a surface the risk of damage to either becomes substantial. Experimental approaches of a pipette to the surface were therefore only performed until a certain drop in current was recorded. The true distance between the pipette and sample is therefore not known, but the above described method of finding $\Delta h_{99\%}$ can still be applied. Supplementary Figure 7c-h show experimental current-distance curves to different surfaces and with pipettes of different size, where the x-axis of each plot has been shifted to match the expected distance from comparison to simulations. All experiments were performed following a procedure, where the pipette was moved vertically at a speed of 100 nm/s towards the surface until a reduction in current of 3% was measured, at which point the pipette was retracted at a speed of 100 nm/s. This procedure was repeated 10 times; 5 times with a pipette potential of +100 mV and 5 times with a pipette potential of -100 mV.

Supplementary Figure 7c and 7d show the experimental approach curves to mica, where the data from each curve is plotted as dots and the solid line is fitted to the entire data. A small pipette was used for the measurements in 7c, and a significant difference between the +100 mV and -100 mV curves is visible, while a larger pipette was used for 6d showing a smaller qualitative difference between the curves. $\Delta h_{99\%}$ can be estimated from both curves, and a value of approximately -5 nm was obtained at 99%. This corresponds to a surface charge density of -34 mC m^{-2}, which is in agreement with values obtained using the scan approach of QSCM.

A mica surface was then vapour coated with APTES to modify the surface charge, and Supplementary Figure 7e and 7f show current-distance curves from a small and a large pipette respectively. APTES molecules will bond to mica and expose positively charged amine groups to the solution, and a positive surface charge is therefore expected[15]. The qualitative difference between curves at +100mV and -100mV was significantly smaller than for mica, and a $\Delta h_{99\%}$ of approximately 0 to 1nm was observed. This corresponds to a mainly neutral surface, which might be the result of an incomplete APTES coverage or an isoelectric point of APTES close to that of the solution pH[16].

Measurements were then performed on a SiO_2 surface, and a qualitative change similar to that of mica was observed. $\Delta h_{99\%}$ was at approximately -2 nm, which corresponds to -16 mC m^{-2}. The negative surface charge density was significantly lower than for mica, which was expected from the material composition[17].

Supplementary Note 4

The effective surface charge density. Charged groups at the surface give rise to a surface charge density. At the same time these charges attract counterions forming a structure termed the electric double layer. Ions inside the double layer are trapped due to a strong influence from the surface, while ions in the bulk solution are mobile and can therefore be described by the Poisson-Boltzmann equation. The difference in ion mobility creates a difference in the traditionally calculated SCD (σ) at the physical surface and the SCD of mobile ions (σ_{eff}). The latter is an experimentally determinable value as it should be linked to the electrokinetic properties near the surface, and it is sometimes referred to as the effective surface charge density[7]. At low surface charge densities and low ionic concentrations Poisson-Boltzmann statistics will be valid even close to the surface linking σ and σ_{eff}[8], but for lipid bilayers of charged lipids at physiological salt concentrations neither conditions is met. In the following the Gouy-Chapman-Grahame-Stern model needed to calculate the SCD of a given lipid bilayer and the extended Poisson-Boltzmann equation needed to correlate it to the effective SCD is described. The electrostatic charge of the lipid bilayer is mainly attributed to the presence of phosphate or amine groups. As an example the deprotonation of an acid group (AH) on the surface can be considered:

$$AH \leftrightharpoons A^- + H^+ \qquad (2)$$

This can be described by the intrinsic dissociation constant given from the site densities of charged (Γ_{A-}) and uncharged, Γ_{AH}, molecules along with the proton activity $[H^+]_s$ at the surface:

$$K_i = \frac{[H^+]_s \Gamma_{A^-}}{\Gamma_{AH}} \qquad (3)$$

The proton activity at the surface can be related to the bulk activity through a Boltzmann distribution assuming a surface potential of ψ_0 compared to the bulk[9]:

$$[H^+]_s = [H^+]_B e^{\frac{-e\psi_0}{k_B T}} \qquad (4)$$

The surface charge density σ is then given by the density of deprotonated groups:

$$\sigma = -e\Gamma_{A^-} = -e\Gamma_{tot} \frac{1}{1+10^{pK_i - pH} e^{\frac{-e\psi_0}{k_B T}}} \qquad (5)$$

Equation (5) gives a relation between the surface charge density and surface potential at the physical surface, while the two entities at the diffuse layer are given by the Grahame equation[10]:

$$\sigma = \sqrt{8C_0 N_A \varepsilon\varepsilon_0 k_B T} \sinh \frac{e\psi_d}{2k_B T} \qquad (6)$$

At the surface a dense layer of favourably packed hydrated counterions forms the Stern layer. This layer is per definition free of charge and acts as a capacitor with a capacitance of:

$$C_S = \frac{\sigma}{\psi_0 - \psi_d} = \frac{\varepsilon_0 \varepsilon_S}{\delta} \qquad (7)$$

Where the outer Stern layer surface is assumed to coincide with the diffuse layer surface, and the capacitance is given as a parallel plate capacitor with a relative permittivity of the interfacial water of ε_S and a thickness equal to the number average of solution ion radius. Solving Supplementary Equations (5-7) provides self-consistent values for the SCD of the three lipids. These values are given in the main text in Table 1. Supplementary Equations (5-7) constitute the traditional model of the double layer, but they rely on the Poisson−Boltzmann equation, which breaks down at high SCD[8]. The effective SCD (σ_{eff}) can be calculated with a model that includes ion size and ion-ion correlations in the double layer[11,12]. Here we have used the extended Poisson-Boltzmann approximation of Attard *et al.*[7], an analytical equation that includes ion correlations, to calculate the effective surface charge in the simple case, where image charges are neglected[8]:

$$\sigma_{\text{eff,lpb}} = \sigma \frac{A}{2s} \sqrt{1 + \frac{e^2\kappa}{16\pi\varepsilon\varepsilon_0 k_B T}(2I + \ln 2)} \qquad (8)$$

This is under the assumption that $\kappa_D \approx \kappa$, $v \approx 1$, which is expected to hold for monovalent ions of reasonable concentration[11]. $\sigma_{\text{eff\_lpb}}$ is the effective surface charge density required for the linear Poisson Boltzmann theory, and:

$$A = \frac{16}{s}\left(-1 + \sqrt{1 + \frac{s^2}{4}}\right) \qquad (9)$$

$$s = \frac{e\sigma}{\kappa k_B T \varepsilon\varepsilon_0} \qquad \text{(S6)}$$

$$I = \frac{1}{2}\left(1 + \frac{2z^3 - 3}{(2z^2-1)^3}\right)\ln 2 + \frac{2 - 2z^3 + z}{2z(2z^2-1)^2} - \frac{1}{2}\left(1 - \frac{2z^2 - 3}{(2z^2-1)^3}\right)\ln(z + z^2) - \frac{\sqrt{z^2-1}}{z}\left(1 + \frac{2z^2+1}{(2z^2-1)^3}\right)\tan^1 \sqrt{\frac{z-1}{z+1}}$$

$$(10)$$

$$z = \sqrt{1 + \frac{s^2}{4}} \qquad (11)$$

$$s_{\text{eff,nl}} = \frac{s_{\text{eff,lpb}}}{1 - \frac{s_{\text{eff,lpb}}^2}{16}} \quad (12)$$

$$s_{eff,nl} = \frac{e\sigma_{\text{eff,nl}}}{\kappa k_{\text{B}} T \varepsilon \varepsilon_0} \qquad (13)$$

$$s_{eff,lpb} = \frac{e\sigma_{\text{eff,lpb}}}{\kappa k_{\text{B}} T \varepsilon \varepsilon_0} \qquad (14)$$

And the inverse Debye length is given as:

$$\kappa = \sqrt{\frac{2cq^2}{\varepsilon\varepsilon_0 k_{\text{B}} T}} \qquad (15)$$

We have used the approach of Attard *et al.*[8], to calculate the effective surface charge density required for both the linear and non-linear Poisson Boltzmann theory. The non-linear Poisson Boltzmann effective surface charge density is used in the main text. The calculations rely on a flat isolated surface, and should therefore be appropriate for the sample surface. The relative permittivity of the Stern layer ε_{S} is set equal to that of the bulk solution for results shown in the main text. The 'true' value is generally considered to be smaller than that of the bulk and is often split into different regions[8], but the value also depend on the charge density of the surface[13]. The value of ε_{S} can be considered the only fitting parameter used in the calculations, but it only affects results for DPPG and as seen in Supplementary Table 4, this is not expected to play a major role. Calculations were performed by solving Supplementary Equations (5-15) simultaneously. A comparison of effective surface charge densities obtained using linear and non-linear Poisson-Boltzmann methods is given in Supplementary Table 5.

Supplementary Note 5

Thermal noise effect on QSCM. Electrical noise will have a significant impact on QSCM as the method relies on a precise measurement of a pico-ampere current. Two unavoidable sources of noise in the ionic current are thermal fluctuations at non-zero temperatures (thermal noise) and the discrete nature of the charge (shot noise). A current of 300 pA totals 1.9×10^9 charges per second, and shot noise will not be of any significance. The root mean square thermal noise can be estimated from the electrical resistance (R) and bandwidth (Δf) of the amplifier[18]:

$$\langle \Delta I \rangle = \sqrt{\frac{4k_{\text{B}} T \Delta f}{R}} \qquad (17)$$

A standard pipette had an inner radius of 15 nm (306 MΩ) and was operated at room temperature. The amplifier bandwidth was 1 kHz resulting in a thermal noise of 0.233 pA or typically 0.0711% of the mean ionic current.

The effect of thermal noise on the measured topography can be estimated as the difference of scanning at setpoints of $0.99I\text{-}\Delta I$ and $0.99I\text{+}\Delta I$. Simulations show the largest effects when imaging a DPPE bilayer (surface charge of 5.3 mC m^{-2}), where the scanning height varies from 14.4 nm to 15.7 nm at a potential of +100mV and from 15.6 nm to 17.1 nm at a potential of -100mV. In the QSCM measurement the standard deviation of surface charge density can be calculated as:

$$\sqrt{\left(5.79\,\frac{\text{mC}}{\text{m}^2\text{nm}}\,\frac{h_{0.99I+\Delta I,V=+100\text{mV}} - h_{0.99I-\Delta I,V=+100\text{mV}}}{2}\right)^2 + \left(5.79\,\frac{\text{mC}}{\text{m}^2\text{nm}}\,\frac{h_{0.99I+\Delta I,V=-100\text{mV}} - h_{0.99I-\Delta I,V=-100\text{mV}}}{2}\right)^2} \quad (18)$$

The thermal noise yields theoretical standard deviation of 5.9 mC m^{-2} (DPPE), 5.4 mC m^{-2} (DPTAP), 3.8 mC m^{-2} (DPPG) and 4.1 mC m^{-2} (mica).

The use of a different setpoint would drastically change the influence of thermal noise. At a setpoint of 98% the standard deviation of surface charge density for a DPPE bilayer would be just 1.0 mC m^{-2}, while at 99.5% it would be 17.7 mC m^{-2}.

The signal to thermal noise ratio scales with the inverse square root of the resistance and the use of a larger pipette might improve the precision.

Supplementary References

1. Atkins, P. & de Paula, J. *Atkins' Physical Chemistry*. (Oxford University Press, New York, 2006).
2. Brown, K. T. & Flaming, D. G. *Advanced micropipette techniques for cell physiology*. (Wiley, New York, 1986).
3. Elsamadisi, P., Wang, Y., Velmurugan, J. & Mirkin, M. V. Polished nanopipets: new probes for high-resolution scanning electrochemical microscopy. *Anal. Chem.* **83,** 671–3 (2011).
4. Perry, D. *et al.* Simultaneous Nanoscale Surface Charge and Topographical Mapping. *ACS Nano* **9,** 7266–7276 (2015).
5. Rheinlaender, J. Lateral Resolution and Image Formation in Scanning Ion Conductance Microscopy. *Anal. Chem.* **86,** 9838–9845 (2015).
6. Caldwell, M., Del Linz, S. J. L., Smart, T. G. & Moss, G. W. J. Method for estimating the tip geometry of scanning ion conductance microscope pipets. *Anal. Chem.* **84,** 8980–4 (2012).
7. Attard, P., Mitchell, D. J. & Ninham, B. W. Beyond Poisson--Boltzmann: Images and correlations in the electric double layer. II. Symmetric electrolyte. *J. Chem. Phys.* **89,** 4358–4367 (1988).
8. Attard, P., Antelmi, D. & Larson, I. Comparison of the zeta potential with the diffuse layer potential from charge titration. *Langmuir* **16,** 1542–1552 (2000).
9. Healy, T. W. & White, L. R. Ionizable surface group models of aqueous interfaces. *Adv. Colloid Interface Sci.* **9,** 303–345 (1978).
10. Israelachvili, J. N. *Intermolecular and surface forces: revised third edition*. (Academic press, 2011).
11. Attard, P. Ion condensation in the electric double layer and the corresponding Poisson-Boltzmann effective surface charge. *J. Phys. Chem.* **99,** 14174–14181 (1995).
12. Ennis, J., Marcelja, S. & Kjellander, R. Effective surface charge for symmetric electrolytes in the primitive model double layer. *Electrochim. Acta* **41,** 2115–2124 (1996).
13. Velikonja, A., Gongadze, E., Iglič, V. K.- & Iglič, A. Charge Dependent Capacitance of Stern Layer and Capacitance of Electrode / Electrolyte Interface. *Int. J. Electrochem. Sci.* **9,** 5885–5894 (2014).
14. Pericet-Camara, R., Papastavrou, G., Behrens, S. H. & Borkovec, M. Interaction between charged surfaces on the poisson - Boltzmann level: The constant regulation approximation. *J. Phys. Chem. B* **108,** 19467–19475 (2004).
15. Lu, Q., Wang, J., Faghihnejad, A., Zeng, H. & Liu, Y. Understanding the molecular interactions of lipopolysaccharides during E. coli initial adhesion with a surface forces apparatus. *Soft Matter* **7,** 9366 (2011).
16. Lin, Y. C., Yu, B. Y., Lin, W. C., Chen, Y. Y. & Shyue, J. J. Site-selective deposition of gold on photo-patterned self-assembled monolayers. *Chem. Mater.* **20,** 6606–6610 (2008).
17. Nishimura, S., Tateyama, H., Tsunematsu, K. & Jinnai, K. Zeta potential measurement of muscovite mica basal plane-aqueous solution interface by means of plane interface technique. *J. Colloid Interface Sci.* **152,** 359–367 (1992).
18. Sarpeshkar, R., Delbruck, T. & Mead, C. A. White Noise in MOS Transistors and Resistors. *IEEE Circuits Devices Mag.* **9,** 23–29 (1993).
